# Notifications of physical, sexual and emotional violence and neglect against children in Brazil, 2011-2019: an ecological time-series study

**DOI:** 10.1590/S2237-96222023000300016.en

**Published:** 2023-11-10

**Authors:** Letícia Regina Morello Sartori, Kaila Andressa dos Santos Oliveira, Kailany Freitas Moura, Pâmela de Oliveira Soares, Valéria Valério Garcia Matos, Sarah Arangurem Karam

**Affiliations:** 1 Universidade Federal de Pelotas, Programa de Pós-Graduação em Odontologia, Pelotas, RS, Brazil; 2 Universidade Federal de Pelotas, Faculdade de Odontologia, Pelotas, RS, Brazil; 3 Universidade Federal de Pelotas, Programa de Pós-Graduação em Epidemiologia, Pelotas, RS, Brazil

**Keywords:** Child Abuse, Child Neglect, Exposure to Violence, Mandatory Reporting, Maltrato a Niños, Negligencia Infantil, Exposición a la Violencia, Notificación Obligatoria, Maus-tratos Infantis, Negligência Infantil, Exposição à Violência, Notificação de Abuso

## Abstract

**Objective:**

To describe the temporal trend of notifications of physical, sexual and emotional violence and neglect against children in Brazil between 2011 and 2019.

**Methods:**

This was an ecological time-series study based on notifications of violence against children aged 0-9 years held on the Brazilian Notifiable Health Conditions Information System. Age-adjusted notification rates were calculated for Brazil as a whole, by national macro-region and by sex. Trends were assessed using Joinpoint Regression.

**Results:**

We analyzed 88,820 notifications of physical violence, 87,141 notifications of sexual violence, 52,359 notifications of emotional violence and 166,664 notifications of neglect. A rising trend was identified for notifications of physical, sexual and emotional violence and neglect for Brazil as a whole and for both sexes. Neglect accounted for the highest rate (95.24 notifications per 100,000 children in 2019). The Northeast and Southeast macro-regions had rising trends for all forms of violence.

**Conclusion:**

Notifications of physical, sexual and emotional violence and neglect showed rising trends in Brazil in the period studied.

## Study contributions

**Main results:**

Notifications of physical, sexual and emotional violence and neglect against children up to 9 years old showed a rising trend in Brazil and its national macro-regions, for both sexes, between 2011 and 2019.

**Implications for services:**

Increasing trends in notifications of violence against children highlight the continued need for capacity building in health services, crucial for early detection, effective prevention and coordinated intervention, taking regional variations into account.

**Perspectives:**

Notification of violence against children by health services requires greater commitment by health workers. Future studies could combine multiple national databases and surveys to increase the accuracy of rates and trends.

## RESUMEN

**Objetivo:**

Describir la tendencia temporal de notificaciones de violencia física, sexual, psicológica y negligencia practicada contra niños en Brasil entre 2011 y 2019.

**Métodos:**

Estudio ecológico de series temporales de notificaciones registradas en el Sistema de Información de Agravamientos de Notificación contra niños (0-9 años). Se calcularon las tasas de notificación ajustadas por edad para Brasil por macrorregión y sexo. Se analizó la tendencia mediante regresión joinpoint.

**Resultados:**

Se incluyeron 88.820 notificaciones de violencia física, 87.141 de violencia sexual, 52.359 de violencia psicológica y 166.664 de negligencia. Se identificó una tendencia ascendente de las notificaciones de violencia física, sexual y psicológica y de negligencia para Brasil y para ambos sexos. La negligencia tuvo la tasa más alta (95,24 notificaciones por 100.000 en 2019).Noreste y Sureste presentaron tendencia creciente en todos los tipos de violencia.

**Conclusión:**

Las notificaciones de violencia contra los niños mostraron tendencia creciente entre 2011 y 2019 en Brasil.

## RESUMO

**Objetivo:**

Descrever a tendência temporal das notificações de violência física, violência sexual, violência psicológica e negligência praticadas contra crianças no Brasil, entre 2011 e 2019.

**Métodos:**

Estudo ecológico de série temporal, sobre notificações de violência contra crianças de 0 a 9 anos no Sistema de Informação de Agravos de Notificação. Taxas de notificação ajustadas por idade foram calculadas para Brasil, macrorregiões nacionais e sexo. Analisou-se a tendência das taxas por regressão *joinpoint*.

**Resultados:**

Foram analisadas 88.820 notificações de violência física, 87.141 de violência sexual, 52.359 de violência psicológica e 166.664 notificações de negligência. Identificouse tendência crescente de notificações de violência física, sexual, psicológica e negligência, para o Brasil e ambos os sexos. Negligência apresentou a maior taxa [95,24 notificações/100 mil crianças (2019)]. Nordeste e Sudeste apresentaram tendência crescente para todas as violências.

**Conclusão:**

Notificações de violência física, sexual, psicológica e negligência contra crianças apresentaram tendência crescente no Brasil, no período.

## INTRODUCTION

Violence against children can be defined as all forms of negligent treatment or ill-treatment that have the potential to cause harm to the health, development and dignity of children.^1^ In particular, such harm can involve a series of damaging effects, both measurable and immeasurable, in both childhood and adulthood.^2^ In childhood, exposure to maltreatment has been associated with physical injuries and adverse mental health outcomes, including cognitive disorders, in addition to the development and presence of internalizing and externalizing psychiatric symptoms.^3^,^4^ Studies with adolescents and adults have shown that exposure to maltreatment in childhood is associated with adoption of risk behaviors, such as smoking, harmful consumption of alcohol and illicit drugs,^5^ in addition to multiple chronic physical and mental health outcomes, such as ischemic heart disease, gastrointestinal diseases and depression.^6^

In quantitative terms, it becomes even more evident how much violence against children is a global emergency.^4^,^7^ A systematic review study estimated that, in 2014, approximately 1 billion children, equivalent to almost half of the world’s population in this age group, had suffered physical, sexual or emotional violence.^7^ Brazilian data reinforce this worrying scenario, considering that in the first half of 2019 alone, approximately 27,000 cases of child abuse were reported to the police in just 12 of Brazil’s 27 Federative Units.^8^

In the context of international initiatives to address child and youth violence, an important milestone in the evolution of social awareness and the institutionalization of rights and protection of children and adolescents in Brazil was the approval of the Child and Adolescent Statute (*Estatuto da Criança e do Adolescente* - ECA): Law No. 8069, dated July 13, 1990.^9^,^10^ Legal mechanisms implemented later, such as the Dial-100 Human Rights service (*Disque-100 Direitos Humanos*)^11^ and the *Menino* *Bernardo* Law (Law No. 13010/2014),^12^ have been developed with the aim of reinforcing the child and youth protection system against exposure to violence. Considering the need to expand and reinforce conventional means of reporting confirmed or suspected cases of violence, such as training police officers and establishing guardianship councils (*conselhos tutelares*), the Notifiable Health Conditions Information System (*Sistema de Informação de Agravos de Notificação* - SINAN) represents an additional frontier to compulsory notification previously established by the ECA.^13^

Suspected or confirmed cases of violence against children began to be registered on the SINAN in 2009. Notwithstanding, its notification became mandatory in all health establishments, both public and private, in 2011.^14^ The SINAN is a component of the Brazilian Violence and Accident Continuous Surveillance System (*Sistema Brasileiro de Vigilância da Violências e Acidentes* - VIVA *Contínuo*), which systematically collects data from the Brazilian health system.^13^ In Brazil, several studies have analyzed temporal trends of notifications of violence against children aged up to 9 years, using SINAN data.^15-22^ Some of these publications have focused on analyzing national data on single years;^15^,^16^ or on data from single Federative Units or their capital cities.^17^,^18^,^21^ We found only one study of data on notification of physical violence against children between 2009 and 2019,^22^ and another study assessing trends in notifications of sexual violence against children up to 14 years old according to sex and age group, between 2009 and 2018.^19^ There is therefore an important relative gap, in the understanding of temporal trend, at national and macro-regional levels, of the four types of violence – physical, sexual, emotional and neglect – against children up to 9 years old. 

When health services perform temporal trend analysis of notifications of violence, this helps to gain knowledge of the magnitude of the problem in the country, in terms of understanding how the notification pattern has been changing, developing health policies and allocating public resources, using an evidence-based approach.^4^

The objective of this study was to describe the temporal trend of notifications of physical, sexual and emotional violence and neglect against children up to 9 years old in Brazil between 2011 and 2019.

## METHODS

### Study design 

This study had an ecological time series design. It was performed using secondary data from the SINAN system, collected and analyzed at a macro-regional level. Our report was prepared according to the guidelines contained in the RECORD Statement (REporting of Studies Conducted Using Observational Routinely-Collected Data).^23^

### Data sources, data collection and eligibility criteria

We analyzed notifications of suspected or confirmed cases of violence against children aged up to 9 years old, retrieved from the SINAN database. Although the ECA defines children as people under 12 years old,^9^ the definition used by the Brazilian Ministry of Health follows the definition recommended by the World Health Organization (WHO), which considers children to be people up to the age of 9.^13^ We included notifications for the following age groups: less than 1, 1 to 4 and 5 to 9 years of age, as per SINAN data. 

We analyzed four types of violence against children, based on Brazilian Ministry of Health definitions, namely: physical violence, sexual violence, emotional violence and neglect.^1,13^ Cases of physical violence are defined as violent acts in which physical force is used to cause harm to the victim, such as, for example, slapping, pinching, pushing or even violence involving firearms.^13^ Sexual violence is defined as a violent act that occurs through the use of power and leads to the loss of the victim’s sexual dignity, such as in episodes of rape or sexual harassment.^13^ Emotional violence is defined as threats, belittling, discrimination, humiliating punishments or other acts involving harm to the victim’s dignity, self-esteem and reputation.^13^ In turn, cases of neglect are considered to be those in which there has been intentional absence of basic care and attending to an individual’s needs.^13^

Notifications without information on the type of violence against children, or that recorded another type of violence (*e.g.*, child labor, human trafficking) were not included. Although SINAN data is available for the period from 2009 to 2021, we only collected and analyzed data from records relating to the period from 2011 to 2019. Records prior to 2011 were not included, given the incipient structuring of the notification network and notification on the SINAN not being mandatory before 2011;^14,15^ notifications made between 2020 and 2021 were not included due to the repercussions of the COVID-19 pandemic on the flow of notifications.^8^

The Tabnet application, developed by the Brazilian National Health System Information Technology Department (DATASUS) and available online (https://datasus.saude.gov.br/informacoes-de-saude-tabnet/), was used to tabulate the absolute number of notifications of suspected or confirmed cases. For this tabulation, active filters, corresponding to each type of violence, the Federative Unit of notification and the sex of the victim, were used for each year of the time series. After tabulation, the information was downloaded in database format, in February 2022. Prior to this stage, four hours of theoretical and practical training were provided with the intention of minimizing potential collection errors. Subsequently, the data was reviewed by another researcher with experience in collecting secondary information, who had not been involved in the initial extraction stage.

The standard Brazilian child population for each year of the time series was obtained from a population projection for 2018, carried out by the Brazilian Institute of Geography and Statistics (*Instituto Brasileiro de Geografia e Estatística* - IBGE).^24^ This projection was developed based on the results of the 2010 Demographic Census, using estimates of the Brazilian population by year, regional division, age group and sex, for the period following 2010.^24^

Notification rates (number of confirmed or suspected cases reported, per 100,000 children), adjusted by age, were calculated using a Microsoft Excel 2016 spreadsheet (Microsoft Corporation, United States), using the direct method.^25^ The absolute number of notifications was obtained for each type of violence (physical, sexual, emotional and neglect), for each year of notification (2011 to 2019), considering the children’s age group (in years: up to 4; 5 to 9). The rate calculation comprised two stages: initially, (i) the annual rates by age group were calculated, using the ratio between the absolute number of notifications and the corresponding child population, multiplied by 100,000; the second stage involved (ii) the adjustment of the rates by the proportion of the standard child population for each age group and year, through simple multiplication and adding up the resulting products. Notification rates at the national level were calculated, considering the standard Brazilian child population. In order to perform stratification according to sex and national macro-region, the Brazilian population for each sex and the child population in each macro-region of Brazil were considered as the standard population, respectively.

### Statistical analysis 

The descriptive analysis of the temporal trend was performed using the Joinpoint Regression Program, version 4.9.0.1.^26^ Joinpoint regression models (inflection points) were applied for physical violence, sexual violence, emotional violence and neglect. Annual percentage change (APC) and respective 95% confidence intervals (95%CI) were estimated to quantify changes in the time series trajectories. Joinpoint regression models perform data trajectory analysis, considering the definition of an initial straight segment by adjusting the series rates.^27^ Next, using the same program, an alternative model was developed with the aim of verifying whether the insertion of break points in the trajectory was statistically significant (α ≤ 5%), for inclusion in the final model.^27^ When statistically significant, the break in the trajectory presents two different straight segments, connected by the inflection point, in which two APC values ​​and respective 95%CI can be presented. In this way, the model evaluates not only changes in the trend, throughout the period that makes up the time series, but also between segments identified by the joinpoint regression model, enabling selection of the best explanatory model for changes in the time series.^27^ Statistical significance was estimated using the permutation test with 4,499 randomly permuted data sets, with subsequent Bonferroni correction, considering the selection of up to one inflection point, according to the software’s default configuration.

Trajectories with positive or negative APC were considered statistically different from zero when α ≤ 5%, being identified, respectively, as rising and falling. Trajectories that did not demonstrate statistically significant differences (p-value > 0.05 and 95%CI containing zero) were considered to have a stable trend.

### Ethical matters 

Tabulations derived from the SINAN are public domain, with unrestricted access, and are presented at an aggregated and anonymized level of information, for Brazil and its regional divisions. Therefore, ethical appraisal was not necessary for the development of this study.

## RESULTS

The study included a cumulative total of 88,820 notifications of physical violence, 87,141 notifications of sexual violence, 52,359 notifications of emotional violence and 166,664 notifications of neglect against children, registered on the SINAN between 2011 and 2019. 

**Figure 1:**
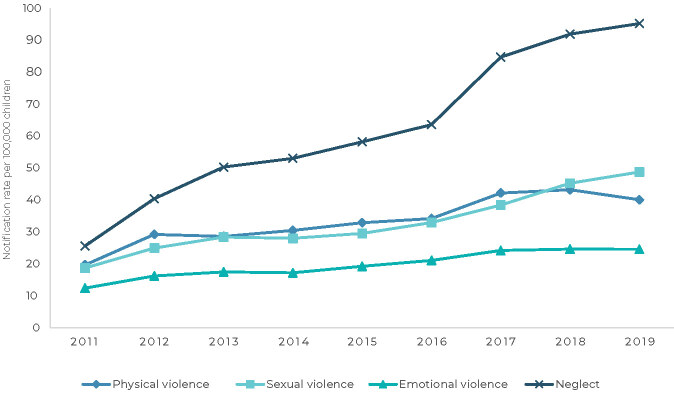
Temporal trends of notification rates of physical violence, sexual violence, emotional violence and neglect committed against children, adjusted by age, Brazil, 2011-2019

At the national level, as shown in Table 1 and illustrated in Figure 1, all types of violence showed rising trends throughout the entire time series assessed. In Brazil as a whole, neglect notification rates had the highest APC in the period (APC = 14.9; 95%CI 11.3;18.6). In 2019, the highest rate (95.2 notifications per 100,000 children) was for notifications of neglect, while the lowest was for notifications of emotional violence (24.6 notifications per 100,000 children). The sexual violence notification rate showed the second highest growth trend (APC = 11.4; 95%CI 8.5;14.4) in the period.

**Table 1:** Temporal trends of notification rates of physical violence, sexual violence, emotional violence and neglect committed against children, adjusted by age, Brazil and national macro-regions, 2011-2019

Violence	Segment	Period		Rate^a^		APC (95%CI)^b^	p-value
		**Initial**	**Final**	**Initial**	**Final**		
**Brazil**							
Physical	1	2011	2019	19.69	40.09	8.0 (5.7;10.5)	< 0.001
Sexual	1	2011	2019	18.73	48.77	11.4 (8.5;14.4)	< 0.001
Emotional	1	2011	2019	12.45	24.60	8.1 (5.9;10.2)	< 0.001
Neglect	1	2011	2019	25.63	95.24	14.9 (11.3;18.6)	< 0.001
**Macro-regions**							
**North**							
Physical	1	2011	2019	16.45	34.41	8.7 (5.4;12.1)	< 0.001
Sexual	1	2011	2019	29.91	54.55	5.2 (2.4;8.0)	0.003
Emotional	1	2011	2019	18.11	29.52	2.5 (-3.4;8.8)	0.349
Neglect	1	2011	2016	8.31	44.55	43.5 (20.6;70.8)	0.004
	2	2016	2019	44.55	44.65	2.7 (-17.7;28.0)	0.758
**Northeast**							
Physical	1	2011	2019	10.65	18.64	7.7 (4.0;11.5)	0.001
Sexual	1	2011	2019	8.48	19.64	10.0 (7.5;12.5)	< 0.001
Emotional	1	2011	2019	5.96	11.47	10.2 (7.5;13.0)	< 0.001
Neglect	1	2011	2019	13.70	56.74	13.4 (6.9;20.4)	0.001
**Midwest**							
Physical	1	2011	2019	23.80	39.50	0.9 (-4.9;7.1)	0.733
Sexual	1	2011	2019	21.77	56.65	9.0 (2.5;15.8)	0.013
Emotional	1	2011	2019	15.01	22.48	-0.8 (-6.5;5.2)	0.752
Neglect	1	2011	2019	49.46	92.52	4.9 (-0.8;11.1)	0.085
**Southeast**							
Physical	1	2011	2019	23.26	49.44	9.9 (4.7;15.3)	0.002
Sexual	1	2011	2019	19.57	53.66	13.4 (11.1;15.8)	< 0.001
Emotional	1	2011	2017	10.27	34.16	23.5 (13.5;34.3)	0.002
	2	2017	2019	34.16	29.39	-6.2 (-28.1;22.3)	0.539
Neglect	1	2011	2019	28.23	87.40	17.8 (12.9;22.8)	< 0.001
**South**							
Physical	1	2011	2013	31.08	53.25	30.9 (-19.2;113.1)	0.200
	2	2013	2019	53.25	63.51	2.1 (-2.1;6.6)	0.238
Sexual	1	2011	2019	29.15	87.06	12.1 (8.5;15.8)	< 0.001
Emotional	1	2011	2019	28.03	35.80	-0.9 (-4.5;2.9)	0.597
Neglect	1	2011	2013	47.32	136.91	65.4 (9.8;149.2)	0.027
	2	2013	2019	136.91	244.73	8.9 (6.7;11.1)	< 0.001

a) Notification rate, standardized per 100,000 children; b) APC (95%CI): Annual percentage change (95% confidence interval).

**Figure 2:**
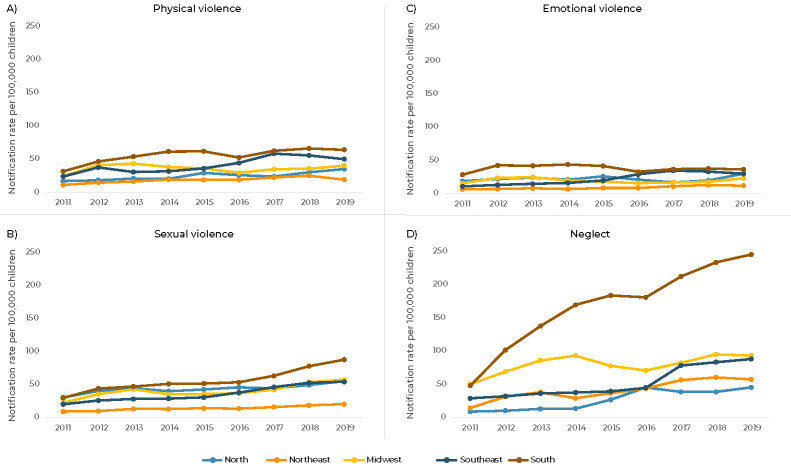
Temporal trends of notification rates of physical violence, sexual violence, emotional violence and neglect committed against children, adjusted by age, Brazil and national macro-regions, 2011-2019

The analysis according to Brazilian macro-regions showed that, at the beginning of the period studied (2011), the Southern region had the highest notification rates for physical violence (31.1 notifications per 100,000 children) and emotional violence (28.0 notifications per 100,000 children), while the Northern region reported the highest rate of sexual violence notifications (29.9 notifications per 100,000 children) and the Midwest region had the highest rate of neglect notifications (49.5 notifications per 100,000 children) (Table 1). At the end of the period studied (2019), the Southern region had the highest notification rates for all types of violence (Figure 2). 

Whereas in the Northern region the sexual violence notification rates (in comparison with the rates of other types of violence) were the highest, both at the beginning (2011) and at the end (2019) of the period studied, in the other macro-regions the highest notification rates in 2011 and 2019 were for neglect (Table 1). 

The Northern region showed rising trends in notifications of physical violence (APC = 8.7; 95%CI 5.4;12.1) and sexual violence (APC = 5.2; 95%CI 2.4;8.0) in the entire period assessed, and rising rates of neglect in the period between 2011 and 2016 (APC = 43.5; 95%CI 20.6;70.8) (Table 1). The Northeast and Southeast regions showed rising trends in notification rates for all types of violence in the period, despite a stable trend in notification rates for emotional violence in the Southeast region, between 2017 and 2019 (APC = -6.2; 95%CI -28.1;22.3). The Midwest region, with the exception of sexual violence, which obtained an APC of 9.0 (95%CI 2.5;15.8), showed a stable trend in other types of violence. A rising trend in sexual violence and neglect notification rates was found in the Southern macro-region throughout the period assessed. 

**Figure 3:**
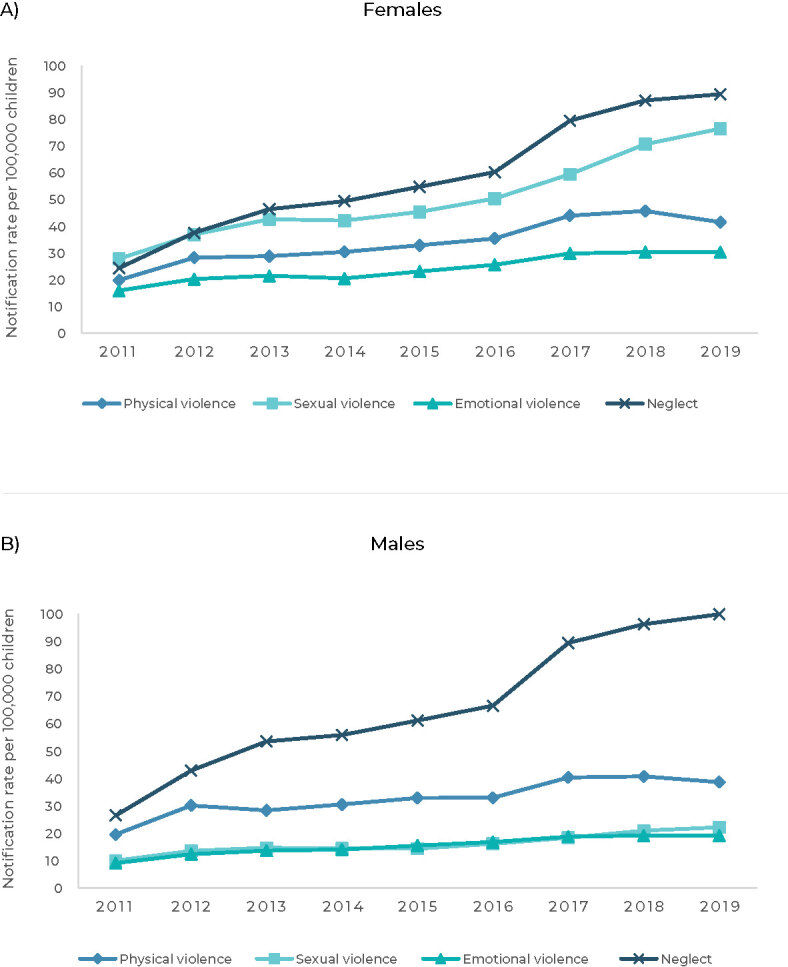
Temporal trends of notification rates of physical violence, sexual violence, emotional violence and neglect committed against children, adjusted by age, according to the sex of the victim, Brazil, 2011-2019

Table 2 presents notification rates and APCs for all types of violence according to the victim’s sex. Rising trends in notifications of all types of violence were found for both sexes (Figure 3). 

**Table 2:** Temporal trends of notification rates of physical violence, sexual violence, emotional violence and neglect committed against children, adjusted by age, according to the sex of the victim, Brazil, 2011-2019

Violence	Segment	Period		Rate^a^		APC (95%CI)^b^	p-value
		**Initial**	**Final**	**Initial**	**Final**		
**Female**							
Physical	1	2011	2019	19.84	41.57	9.1 (6.3;11.9)	< 0.001
Sexual	1	2011	2019	27.84	76.56	12.3 (9.4;15.2)	< 0.001
Emotional	1	2011	2019	15.94	30.31	7.9 (5.8;10.0)	< 0.001
Neglect	1	2011	2019	24.38	89.38	15.1 (11.7;18.5)	< 0.001
**Male**							
Physical	1	2011	2019	19.55	38.67	7.0 (4.8;9.2)	< 0.001
Sexual	1	2011	2019	10.00	22.20	8.9 (5.9;11.9)	< 0.001
Emotional	1	2011	2019	9.10	19.15	8.3 (6.0;10.6)	< 0.001
Neglect	1	2011	2019	26.50	100.67	14.9 (11.1;18.7)	< 0.001

a) Notification rate, standardized per 100,000 children; b) APC (95%CI): Annual percentage change (95% confidence interval).

The highest notification rates for physical, sexual and emotional violence, in 2011 and 2019, were found among females. In contrast, the highest notification rates (initial and final) of neglect were found for male victims. The highest APCs for physical violence (APC = 9.1; 95%CI 6.3;11.9), sexual violence (APC = 12.3; 95%CI 9.4;15.2) and neglect (APC = 15.1; 95%CI 11.7;18.5) were for female children (Table 2). 

## DISCUSSION

The results found demonstrated that, between 2011 and 2019, notifications of physical, sexual and emotional violence and neglect against children aged up to nine years showed rising trends in Brazil. The highest notification rates were found for neglect, while the lowest were for emotional violence. At the end of the period (2019), the Southern macro-region had the highest notification rates for all types of violence against children, despite a rising trend only in notification rates for sexual violence and neglect in that macro-region. In contrast to the other macro-regions, where neglect had the highest rate, in the Northern region sexual violence had the highest notification rate. A rising trend in notifications of sexual violence was found in all Brazilian macro-regions. The Northeast and Southeast regions showed rising trends for other types of violence. Considering the sex of the victim, a rising trend was found in notifications for children of both sexes, in all types of violence, despite different notification patterns being identified for females and males.

In Brazil, as in other Latin American countries, social norms consider physical, emotional and verbal aggression against children to be “educational and disciplinary strategies”.^28^ This culture of corporal and verbal punishment, normalized as a parental educational practice, may be related to the passive view of health professionals and, consequently, to underreporting, especially in less serious cases of physical and verbal violence. However, cases of neglect and sexual violence tend not to be viewed passively by health professionals – a factor that could explain the high rate of notifications of neglect and rising trends in notifications of sexual violence found in this study. Similarly, a previous study found that 47.5% of notifications of violence against children up to 9 years old recorded on the SINAN in 2011 were due to neglect or abandonment.^16^ Also in 2011, notifications of emotional violence accounted for only 25.2% of total violence against children.^16^ A possible reason for the low notification rates found for emotional violence centers on the SINAN recommendations that only the main type of abuse suffered should be notified, while emotional violence, in general, occurs concomitantly with other types of aggression.^13^,^17^

Considering the results found for macro-regions, also in 2011, the Southern macro-region had the highest rates for physical and emotional violence, and in 2019 it had the highest notification rates for all types of violence, despite a rising trend only having been found for sexual violence and neglect. A previous study, which used national data recorded on the SINAN during 2010, found that the three states that make up the Southern macro-region had some of the highest notification rates of violence against children.^15^ Furthermore, the Northeast and Southeast regions showed rising trends in notifications in the time series assessed, reinforcing previous findings once more.^15^,^16^,^22^ This could possibly be explained by the history of the establishment of the notification network,^15^ as well as the growing concentration of health establishments in the Northeast, Southeast and South of Brazil and, consequently, wider access to and use of health services.^29^ A study developed using sexual violence data held on the SINAN for the state of Santa Catarina (southern macro-region) found that 46.7% of the variation in the number of notified cases of sexual violence was explained by the number of referral centers for cases of violence, between 2009 and 2019.^21^

Furthermore, all Brazilian macro-regions showed a rising trend in notifications of sexual violence. Corroborating the results of this study, previous research that assessed Brazilian notifications of sexual violence found year by year increases in the period from 2010 to 2018.^19^ In addition, only the Northern region was found to have a higher notification rate for sexual violence than for neglect. This pattern was also found by previous studies, conducted using notification data from the Northern region cities of Manaus^18^ and Belém,^17^ which may indicate greater underreporting of other types of violence on the SINAN in that region; or, even, a better established structure for tracking and caring for victims of sexual violence in the Northern macro-region.^18^

It is consistently reported in the literature that female children tend to be the majority of victims of sexual violence, while male children tend to be the majority in cases of physical violence and neglect.^15^,^16^,^19^,^22^ This characteristic of notifications appears to be strongly related to gender stereotypes, which influence vulnerability, experiencing and reporting of violence against boys and girls.^4^,^28^ Notwithstanding, our study found that female victims had the highest initial and final rates, not only for sexual and emotional violence but also for physical violence, with male children having the highest notification rates for neglect. Furthermore, in this study, positive APC was statistically significant for both sexes. This result is corroborated by previous studies, which also found significant increases in notifications of sexual violence against boys and girls, reinforcing the need to target campaigns to prevent violence against them.^19^,^20^

In addition to the conclusions of this study, it is essential to highlight its weaknesses. Firstly, we should mention the possibility of underreporting of violent episodes against children. It is always important to keep in mind that the number of incidents of violence captured by the health system is mainly made up of cases that, in some way, require physical or mental health care, as these are often recurring cases.^2^,^15^ Secondly, we did not assess the completeness of the data records used, potential duplication of records or concomitant analysis comparing them with other databases (i.e. police and child protection service records) that could increase accuracy as to the rising national trends for these types of violence. Therefore, the results of this research should be interpreted with caution, since the rising trends found may be a consequence of (i) the real increase in cases, (ii) better interpretation of cases and increased notifications by health professionals, or even, (iii) the combination of both. Finally, we only performed analyses by gender and national macro-region; other characteristics, such as race/skin color and ethnicity, were not included due to the massive presence of blank or unknown data – which hinders intersectional analysis and interpretation of results.

The strengths of this study, in turn, include the evaluation of the 9-year time series of notifications of child abuse in Brazil, which makes this study relevant for an initial understanding of the pattern of notifications after almost a decade of mandatory notification by public and private health services. Additionally, studies developed with data collected during the routine of health services are extremely useful, in order to promote planning and monitoring policies, to break the cycle of violence against children. Considering all the harmful effects that exposure to violence has on individuals’ lives, reporting violence against children and adolescents is part of comprehensive care for victims and constitutes a legal, moral, ethical and humanitarian responsibility of health professionals and health center managers.^1^,^4^,^9^

In conclusion, this study demonstrated that notifications of physical violence, sexual violence, emotional violence and neglect against children showed rising trends in Brazil between 2011 and 2019. The highest notification rates were found for neglect; with the exception of the Northern region, which, in 2019, had the highest sexual violence notification rate. The Northeast and Southeast macro-regions showed a rising trend for all types of violence analyzed. Notifications of sexual violence showed rising trends in all five major Brazilian regions. Finally, statistically significant increases were found in the annual percentage change in notification rates for all types of violence committed against children in Brazil. 
